# Building Learning Health Systems to Accelerate Research and Improve Outcomes of Clinical Care in Low- and Middle-Income Countries

**DOI:** 10.1371/journal.pmed.1001991

**Published:** 2016-04-12

**Authors:** Mike English, Grace Irimu, Ambrose Agweyu, David Gathara, Jacquie Oliwa, Philip Ayieko, Fred Were, Chris Paton, Sean Tunis, Christopher B. Forrest

**Affiliations:** 1 Nuffield Department of Medicine, University of Oxford, Oxford, United Kingdom; 2 KEMRI-Wellcome Trust Research Programme, Nairobi, Kenya; 3 Department of Paediatrics, College of Health Sciences, University of Nairobi, Nairobi, Kenya; 4 Dean, College of Medicine, College of Health Sciences, University of Nairobi, Nairobi, Kenya; 5 Center for Medical Technology Policy (CMTP), Baltimore, Maryland, United States of America; 6 Children's Hospital of Philadelphia, Philadelphia, Pennsylvania, United States of America

## Abstract

Mike English and colleagues argue that as efforts are made towards achieving universal health coverage it is also important to build capacity to develop regionally relevant evidence to improve healthcare.

**Summary Points**

- Achieving universal coverage that supports high-quality care will require that health systems are designed to integrate the delivery of health services with the generation of new knowledge about the effectiveness of these services.
- System strengthening and research will need to be better integrated to achieve this in low- and middle-income countries (LMIC) so that changes in coverage, quality, and impact are measured, costs are contained, and health systems are responsive to users’ needs and concerns.
- In high-income countries, learning health systems (LHS) are emerging to meet similar needs. The LHS vision aspires to engage policy makers, researchers, service providers, and patients in learning that uses and strengthens routinely collected data to conduct pragmatic, contextually appropriate research, promote rapid adoption of findings to improve quality and outcomes, and promote continuous learning.
- Although there are significant challenges, we should begin to develop LHS in LMIC for their immediate and longer term benefits and to avoid having to retrofit health systems with the capability to promote learning at a later date and even greater cost.
- A global coalition on how to build LHS effectively that shares accumulating learning could enable such a strategy.

## Background

Achieving broad coverage of health interventions and optimizing health outcomes is a major challenge for low- and middle-income countries (LMIC). In many areas, we are faced by striking inadequacies in the evidence base, evidence gaps are being tackled at too slow a pace and too high a cost [1], and traditional vertical programmes and academic research initiatives are failing to impact on broader health systems. There are further difficulties generalizing the evidence from efficacy trials with restrictive enrolment criteria in high-income countries (HIC) to the realities of LMIC, where diagnostic, therapeutic, and skilled human resources are limited. Furthermore, even when good evidence is produced, it can take many years for clinicians and policy makers to act upon new knowledge [2,3]. These problems are particularly acute in LMIC that have the greatest burden of health care need and limited resources with which to address those needs. Health systems may therefore waste resources and deliver poor outcomes.

Here we argue for a deliberate and strategic emphasis on building learning health systems (LHS) within LMIC capable of identifying those interventions and implementation strategies that work in routine contexts and of improving quality consistently across the health system. The Institute of Medicine describe a learning health care system as one “that is designed to generate and apply the best evidence for the collaborative health care choices of each patient and provider; to drive the process of discovery as a natural outgrowth of patient care; and to ensure innovation, quality, safety, and value in health care” [4]. Critically, the focus is on patient-important outcomes, solving practical problems of service delivery, generating new evidence where required by research including the full range of patients encountered in routine practice, and rigorous evaluation of intervention effectiveness.

The LHS approach thus deliberately combines concerns for improving quality through a focus on appropriate infrastructure, resources, and understanding the process and outcomes of health care with the service delivery and implementation research needed to extend effective coverage in varying contexts within LMIC health systems. However, it goes beyond this to encompass tests of intervention effectiveness through conduct of much-needed pragmatic clinical trials [5,6]. Indeed, LHS provide an opportunity for conducting trials that are efficiently integrated within routine care. Further, the high-quality data that pragmatic trials, implementation studies, and quality improvement all demand mean attention in LHS is focused on the need to optimize health information systems that are also critical to effective performance measurement and management.

Developing LHS in LMIC will have to be undertaken deliberately and carefully. Producing better data during routine care will need to reduce, not increase, the reporting tasks of scarce LMIC health workers. To promote participation in research and other forms of learning, to ensure research is relevant to patient needs, and to promote uptake of findings, policy makers, practitioners, and patients in routine settings must become partners in the learning enterprise. This contrasts with the fragmented approach to system strengthening, implementation of essential interventions, research, and quality improvement currently found in many LMIC that is unlikely to produce a health system capable of continuous improvement, and as HIC are finding, there are major costs of trying to retrofit such learning into health systems.

### Lessons from High-Income Settings

Networks of facilities and practitioners are at the heart of learning systems. Some with the greatest longevity (such as the United States Children’s Oncology Group [7]) rely to a large extent on the willingness of health workers in practice to devote a portion of their limited time to the learning enterprise. They do so because they see value both in the effort, which provides them with new ways to improve the health and lives of their patients, and in being part of a stimulating community of like-minded colleagues.

Similarly, secondary analysis of routinely collected clinical data supports an increasing number of large comparative effectiveness evaluations. Embedding randomized trials in clinical practice may dramatically reduce the cost of trials [1] and increase the speed of research requiring high levels of internal validity [8]. Research within practice settings also helps prime the policy and clinical communities to implement the findings, greatly shortening the time from study to patient impact.

LHS that emerged in some HIC have their origins in smaller, often geographically or thematically linked networks, a trajectory that may be an early model for LMIC. Some successful examples include the Group Health Cooperative in Seattle, Washington, US, [9] and the theme-specific PEDSnet that spans eight US paediatric health systems, 22 states, and 4.7 million children [10]. LHS may also be part of broader, national strategies such as the substantial investments being made in the US National Patient-Centered Outcomes Research Network (PCORnet) [11] or England’s Academic Health Science Networks [12]. Some of the broad principles of LHS are to advance patient health through (i) creating a network of engaged and highly motivated stakeholders who get involved in all aspects of the system, including its design, operation, and governance; (ii) enabling clinical staff to use information tools (ultimately electronic health records) efficiently integrated into their routine work so that clinical data are entered only once and repurposed many times for research, quality improvement, and wider health system performance monitoring (the data-in-one principle); (iii) conducting rapid and efficient clinical and population health research using observational and experimental methods; (iv) fostering implementation of evidence into routine clinical care to improve quality and outcomes, and (v) shared, continuous learning through the creation of common resources [4].

### Challenges and Opportunities for LHS in Low-Income Settings

#### Research partnerships and capacity

Although research funding has increased in LMIC, a small proportion comes from national governments, especially in low-income countries. Much health research is therefore globally driven, illness specific, explanatory, short term, or small scale. Although there are some substantially funded health programmes, these may be poorly integrated into the wider health or research system. The creation of collaborative communities, a necessary first step, will require the development of long-term partnerships between governments, research funders (including international foundations and global development donors), policy makers, health care providers, patients, and communities. These partnerships promote research that matters for policy and practice, sharing of resources, and the active participation of all partners, helping strengthen trust and capacity over time.

Research leadership will also be required. The need to expand research capacity is recognized globally and requires active engagement of the medical education community [13] to produce health workers familiar with the principles of data-driven quality improvement, experimental methods, and evidence-based medicine. This is particularly important in LMIC, where much care is provided by practitioners with limited training and experience often operating in relative isolation, something LHS may also address. The LHS model also provides a coherent way to ensure capacity building in nontraditional fields such as health informatics, statistics, patient engagement, implementation science, and health service evaluation. Professionals in these areas will likely be critical to the functioning of future health systems, and their numbers should expand with rapid growth in tertiary education in LMIC.

#### Investment

Creating learning networks will require essential investments and political will. In low-income countries, recognition of the need for evidence-informed policy is gaining considerable traction, and a number of LMIC are developing capacity to conduct and use systematic reviews. This provides an opportunity for researchers to engage with health system leaders, something they have often found difficult. Concern for better information on health system performance, accountability to citizens, and an appreciation that technology is changing the health system landscape provide other opportunities. However, there are perhaps few robust existing mechanisms that promote patient engagement in planning care or research with power imbalances related to social and educational hierarchies. However, responsiveness and accountability represent a global discourse [14] that could support development of patient, provider, and researcher engagement.

#### Information infrastructure

A number of major technical or institutional developments need to be fostered. Electronic health records (EHR) that might support collection of standardized data as part of routine care across multiple facilities are just emerging in many LMIC. Nonetheless, many countries are experiencing rapid growth in their information and communication technology (ICT) sectors, with investments being made in improving internet and mobile connectivity, even in rural areas. The success of mobile phone-based business applications and a hunger for the efficiencies and effect on equity of access that eHealth may bring are all providing an impetus to extend EHR.

An emerging, poorly regulated sector made up of multiple small EHR system vendors is, however, a challenge. Leadership that promotes common data standards within systems as part of broader informatics standards will be a critically important accelerator of success and long-term interoperability. This approach is already yielding system-wide data within the field of HIV in Africa [15]. Moreover, quick action in this area could prevent later interoperability problems such as those being faced by HIC with extensive legacy systems.

However, the costs and technical challenges of national EHR introduction in HIC, harsh physical environments in some areas, and competing priorities for investment suggest a measured approach in LMIC. As a relatively small number of conditions result in much of the morbidity and mortality, rapid results may be delivered by initially more simple and focused routine data collection systems. By developing common data frameworks, broad coverage may be possible, such as with the Mini-Sentinel system [16]. Early examples of the potential for such approaches also exist in African settings [17], and a further example is presented in Box 1.

**Box 1. Building the Data Needed for Learning: A Clinical Network in a Low-Income Setting**

In Kenya, a collaborative partnership spanning researchers, policy makers, professional associations, and providers is working to establish a paediatric clinical information network across 14 hospital sites. Common data elements derived from improved paper-based records on 2,500 inpatient children per month are collated daily at low-cost using nonproprietary software [18]. Although the quality of information within records was initially modest or poor, working with the data and engaging clinicians in seeing their value considerably and rapidly improved data quality. Advances in methods to handle missing data, use of comparative observational analysis methods that reduce risks of bias (for example, propensity score adjustment or interrupted time series methods), and a focus on hard outcomes such as mortality and on conduct of pragmatic randomized trials within the network are all aims to support meaningful analysis of routine data. In our Kenyan example, data are now therefore beginning to inform efforts to improve adoption of evidence-based practices and quality of care and inform research design.

#### Data management and governance

To support both patient care and learning will require management of big data resources. While new to the health sector for many countries, telecommunication companies, banks, and even government tax offices have demonstrated this type of capacity in LMIC. Beyond technology, many countries may also rapidly need to develop appropriate data governance policies, appropriate ethical guidance, and “one-stop,” central institutional review boards for multisite studies. Here efficient progress may be made by learning from international practice, noting that this is still evolving even in high-income settings [19,20]. These endeavours need to be combined with major efforts to learn from and educate the public, health care providers, managers, and ethical review boards in a deliberative and dynamic process to build trust amongst all parties. This discourse needs to articulate that all have a role to play in contributing to the common good of improved health care, moving on from the more typical discourse focused on the researcher and the researched [21].

### A Way Forward

To embed learning in routine clinical practice will require the formation of organizational entities in which, unlike conventional projects that shutdown once the analyses are disseminated, a learning partnership is designed for long-term sustainability. Establishing a coalition of highly committed stakeholders built around existing or nascent policy, research, and practice networks might allow the most rapid progress towards successful development of broader LHS. We illustrate the potential advantages of embedding even a well-integrated clinical trial into a LHS in Box 2.

**Box 2. Illustrating the Added Value of Conducting a Pragmatic Trial within an LHS**

Although multiple large randomized controlled trials provided moderate certainty of effect estimates that oral antibiotic therapy was noninferior to injectable therapy for one category of pneumonia, a change in policy was rejected in Kenya [22]. Concerns were that trial patients were not representative of the local reality in which comorbidity and fatal outcomes were felt to be common. These, and similar discussions with policy makers in Kenya in 2005, 2010, 2013, and 2014 ([22,23] and unpublished observations), have emphasized the need for engagement of stakeholders in deciding on the need for trials and provided the rationale for a local pragmatic trial [24]. Results supported noninferiority of oral treatment. The engagement with stakeholders then informed a change in national treatment recommendations prior to formal study publication and identified priorities for new research to monitor the effects of implementation in rural populations with poorer access to care.

Although well embedded in the local policy and practice environment, this trial did not benefit from many of the efficiencies that might be gained within a formal LHS. No data on the clinical effectiveness of the standard antibiotic regimens existed, so the trial had to be preceded by a preliminary assessment of outcomes with treatment that was standard of care [24]. Questions asked in the policy debate on the conclusion of this trial that could not be supported by data concerned the availability of pulse oximetry to help gauge disease severity, patients’ preferences for oral treatment, and potential risks associated with outpatient care in communities that travel large distances on foot, making reattendance for review or in case of deterioration problematic. A LHS might provide some of these data or at least provide the framework for further efficiently conducted research to obtain it. Furthermore, a LHS would provide the mechanism for monitoring and improving implementation of the new policy with ongoing monitoring of the effectiveness after implementation and tracking quality of care—all difficult at present. [2]

Taking the examples outlined in Boxes 1 and 2, we can begin to see how LHS might realistically emerge initially in networks of modest size. Concern with the continuing high burden of mortality and morbidity from childhood pneumonia could, for example, drive multiple stakeholders including government and researchers to consider the challenge holistically. Efforts could be made to define what data could be derived from high-quality consultations and an ability to track treatments and outcomes. Engagement with providers and those responsible for the health information system might allow some critical data to be collected routinely within such a network. Observational data might be used to foster simple improvements in care (e.g., adherence to guidelines) and examine outcomes in detail. Engagement with providers and families might highlight problems with service delivery and help explain existing outcomes. This, with input of all parties, might suggest the need to test innovations in service delivery (e.g., use of short-stay observation units) or alternative therapeutic strategies (e.g., continuous positive airway pressure [CPAP]) that best fit the context and problem and using optimal study designs including pragmatic randomized controlled trial (RCT) where appropriate. While this hypothetical case focuses on pneumonia, it is important that such learning becomes embedded in routine work. Any substantive changes to health information systems, for example, should be based on long-term data needs. Core data requirements should be disseminated, enabling those providing electronic medical records to rapidly adopt such emerging standards in line with regulation that promotes interoperability. Thus, learning within a network might help establish quality improvement and inform policy on service delivery, treatment, and health information systems on a wider scale. Lessons from the learning process can be rapidly applied to other clinical concerns within the network and shared across networks. Slowly, capacity and a culture that enable more rapid learning and health system improvement might be built.

Indeed, as such model LHS are developed, there is potential to develop a network of emerging LHS that would inform efficient spread of concepts and practice more widely. In addition, LHS could form the basis of collaboration on transnational research, for example, partnering to conduct multicountry therapeutic trials, evaluation of diagnostics, strategies for patient engagement, and developing and testing enhancements to health information systems or alternative models for delivering care for chronic diseases.

### Conclusions

LHS, even in early forms, hold great promise as a means to provide timely, relevant research that is efficiently conducted and implemented to improve quality of patient-centred care and health. They offer the potential of building capacity for research, implementation, patient engagement, and evidence-informed policy making in line with best practice [25]. With a focus on common data and longer-term interoperability, they have the potential to foster growth of health systems that leverage to best future advantage the benefits of big data for patients, providers, managers, and researchers. A critical first step is building substantive, long-term collaborations between decision makers, researchers, networks of providers, and funders in which funders support but do not dominate priority setting and decision making. LMIC have potentially the most to gain from LHS. As efforts are made towards achieving universal health coverage, it will also be important to build the capacity for health systems to develop regionally relevant evidence to improve health care. There should be concerted efforts made to promote the development of LHS and integrate them into a global network of learning sites.

## Abbreviations

CPAP: continuous positive airway pressure
EHR: electronic health record
HIC: high-income country
ICT: information and communication technology
KEMRI: Kenya Medical Research Institute
LHS: learning health system
LMIC: low- and middle-income countries
PCORnet: United States National Patient-Centered Clinical Research Network
RCT: randomized controlled trial
